# Morphine hydro­chloride anhydrate^1^

**DOI:** 10.1107/S1600536812046405

**Published:** 2012-11-17

**Authors:** Thomas Gelbrich, Doris E. Braun, Ulrich J. Griesser

**Affiliations:** a Institute of Pharmacy, University of Innsbruck, Innrain 52c, 6020 Innsbruck, Austria

## Abstract

In the title mol­ecular salt [systematic name: (5α,6α)-7,8-didehydro-4,5-ep­oxy-17-methyl­morphinan-3,6-diol hydro­chloride], C_17_H_20_NO_3_^+^·Cl^−^, the conformation of the morphinium ion is in agreement with the characteristics of the previously reported morphine forms [for example, Gylbert (1973 ▶). *Acta Cryst.* B**29**, 1630–1635]. In the crystal, the cations and chloride anions are linked into a helical chain propagating parallel to the *b*-axis direction by N—H⋯Cl and O—H⋯Cl hydrogen bonds. The title salt and the morphine monohydrate [Bye (1976 ▶) *Acta Chem. Scand.* **30**, 549–554] display very similar one-dimensional packing modes of their morphine components.

## Related literature

For related structures, see: Guguta *et al.* (2008 ▶); Gylbert (1973 ▶); Mackay & Hodgkin (1955 ▶); Bye (1976 ▶); Wongweichintana *et al.* (1984 ▶); Lutz & Spek (1998 ▶); Scheins *et al.* (2005 ▶). For hysdrogen-bond motifs, see: Bernstein *et al.* (1995 ▶); Etter *et al.* (1990 ▶). For a description of the Cambridge Structural Database, see: Allen (2002 ▶). For the program *XPac*, see: Gelbrich & Hursthouse (2005 ▶) and for the corresponding *XPac* dissimilarity index, see: Gelbrich *et al.* (2012 ▶).
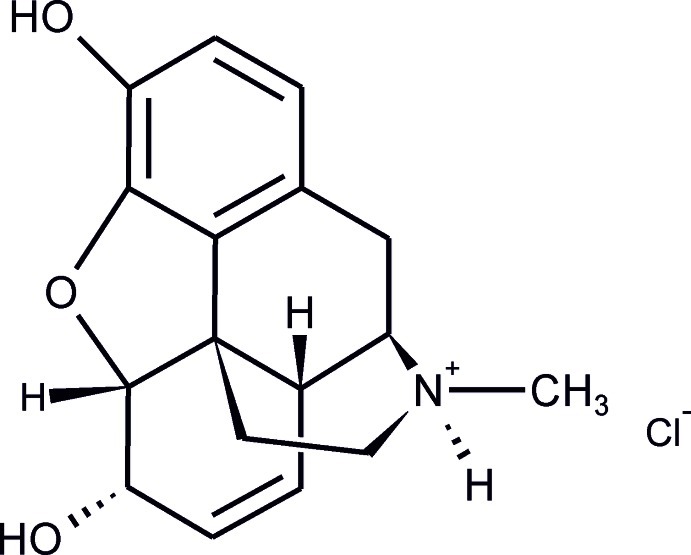


## Experimental

### 

#### Crystal data

C_17_H_20_NO_3_^+^·Cl^−^
*M* *_r_* = 321.79
Orthorhombic, 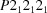

*a* = 7.3504 (2) Å
*b* = 12.8524 (5) Å
*c* = 16.0372 (5) Å
*V* = 1515.04 (9) Å^3^
*Z* = 4
Mo *K*α radiation
μ = 0.27 mm^−1^
*T* = 173 K
0.20 × 0.20 × 0.20 mm

#### Data collection

Oxford Diffraction Xcalibur (Ruby, Gemini ultra) diffractometer
Absorption correction: multi-scan (*CrysAlis PRO*; Oxford Diffraction, 2003 ▶) *T*_min_ = 0.982, *T*_max_ = 1.000
7406 measured reflections
2971 independent reflections
2803 reflections with *I* > 2σ(*I*)
*R*_int_ = 0.024

#### Refinement

*R*[*F* ^2^ > 2σ(*F* ^2^)] = 0.027
*wR*(*F* ^2^) = 0.069
*S* = 1.04
2971 reflections
229 parameters
3 restraints
H atoms treated by a mixture of independent and constrained refinement
Δρ_max_ = 0.18 e Å^−3^
Δρ_min_ = −0.15 e Å^−3^
Absolute structure: Flack (1983 ▶), 1245 Friedel pairs
Flack parameter: 0.02 (5)

### 

Data collection: *CrysAlis PRO* (Oxford Diffraction, 2003 ▶); cell refinement: *CrysAlis PRO*; data reduction: *CrysAlis PRO*; program(s) used to solve structure: *SHELXL97* (Sheldrick, 2008 ▶); program(s) used to refine structure: *SHELXL97* (Sheldrick, 2008 ▶); molecular graphics: *XP* in *SHELXTL* (Sheldrick, 2008 ▶) and *Mercury* (Macrae *et al.*, 2008 ▶); software used to prepare material for publication: *publCIF* (Westrip, 2010 ▶).

## Supplementary Material

Crystal structure: contains datablock(s) I, global. DOI: 10.1107/S1600536812046405/lx2271sup1.cif

Structure factors: contains datablock(s) I. DOI: 10.1107/S1600536812046405/lx2271Isup2.hkl

Additional supplementary materials:  crystallographic information; 3D view; checkCIF report

## Figures and Tables

**Table 1:** Hydrogen-bond and short-contact geometry (Å, °) *Cg*1 is the centroid of the C1–C4/C12/C11 benzene ring.

*D*—H⋯*A*	*D*—H	H⋯*A*	*D*⋯*A*	*D*—H⋯*A*
O1—H1*O*⋯Cl1	0.82 (1)	2.35 (1)	3.1585 (14)	173 (2)
O3—H3*O*⋯Cl1	0.83 (1)	2.32 (1)	3.1416 (13)	168 (2)
N1—H1*N*⋯Cl1^i^	0.93 (1)	2.15 (1)	3.0626 (15)	169 (2)
C17—H17*C*⋯O3^ii^	0.98	2.38	3.278 (2)	153
C14—H14⋯O2^i^	1.00	2.60	3.2149 (19)	120
C2—H2⋯*Cg*1^iii^	0.95	2.71	3.638 (2)	165

Symmetry codes: (i) 
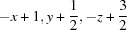
; (ii) 
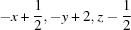
; (iii) 
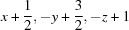
.

**Fig. 1.**
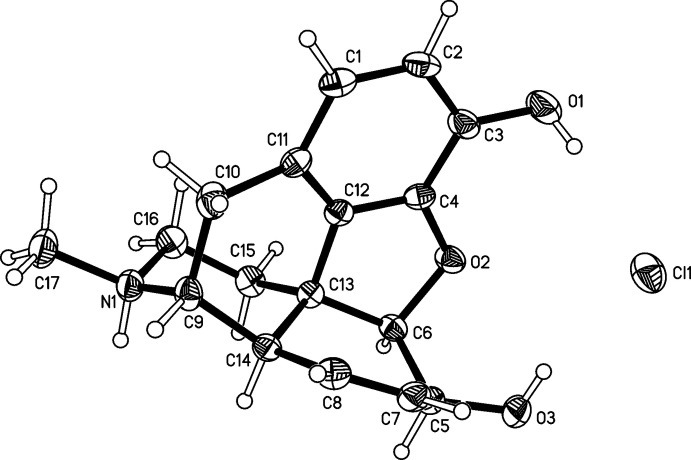
Asymmetric unit of the title compound. Displacement ellipsoids are drawn at the 50% probability level, with hydrogen atoms shown as spheres of arbitrary size.

**Fig. 2.**
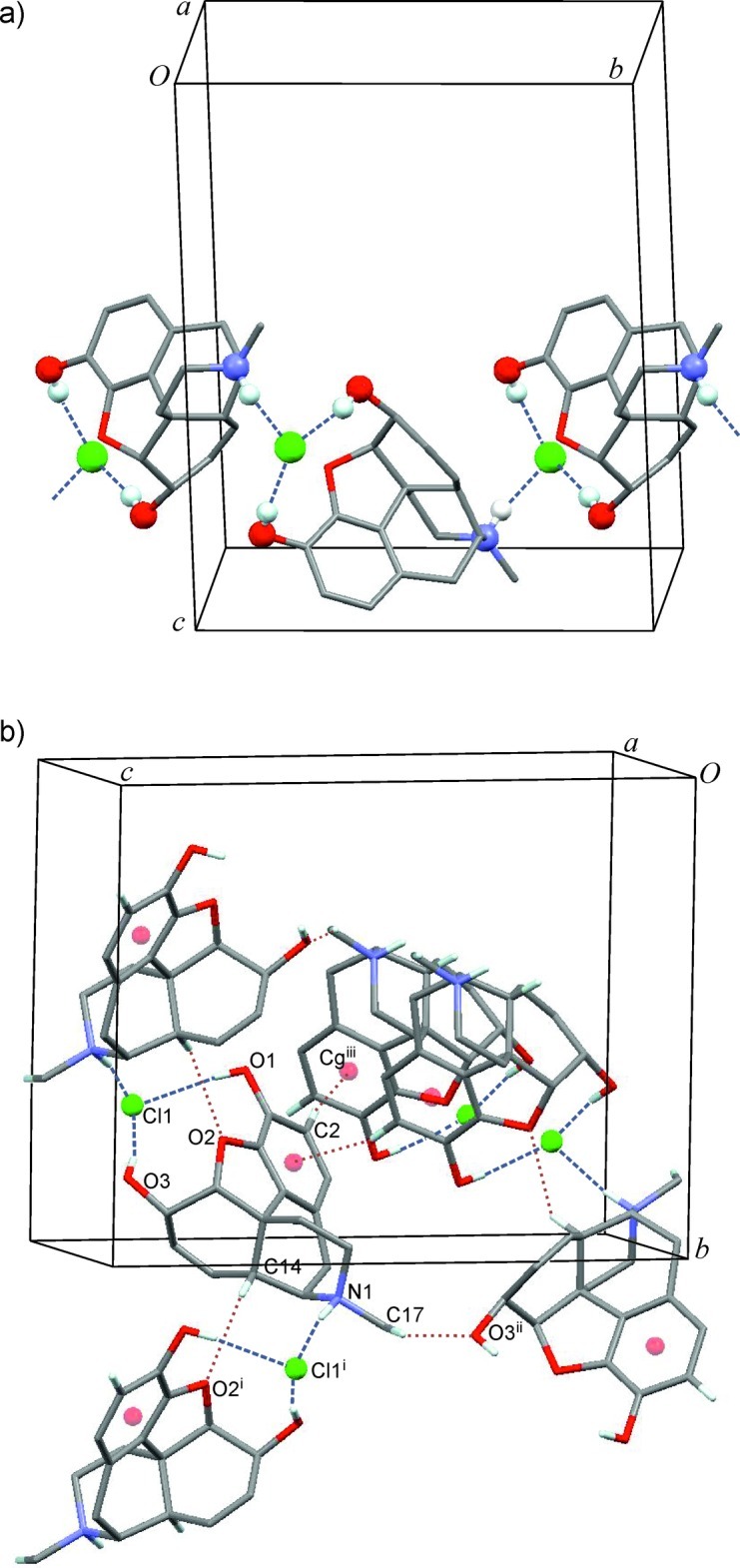
a) The hydrogen–bonded chain structure of the title salt. H, O and Cl atoms involved in hydrogen bonding are are drawn as balls and O—H···Cl and N—H···Cl interactions are drawn as dotted lines. H atoms non–participating in hydrogen–bonding were omitted for clarity. b) Impterplay of classical hydrogen bonds (broken lines) and short O—H···X contacts (dotted lines); symmetry codes: (i) -x + 1, y + 1/2, -z + 3/2; (ii) -x + 1/2, -y + 2, z - 1/2; (iii) x + 1/2, -y + 3/2, -z + 1.

**Fig. 3.**
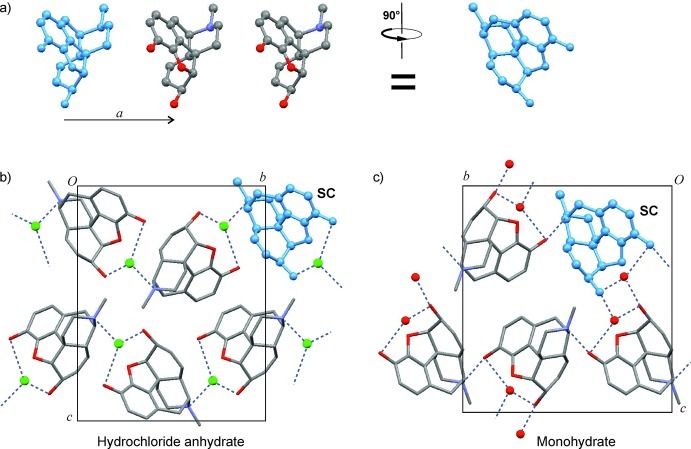
Common one-dimensional supramolecular construct (SC) of the title structure and the morphine monohydrate (Bye, 1976). a) Stack of molecules related by translation symmetry, viewed perpendicular and parallel to the stacking vector (which lies parallel to the a–axis in both structures); crystal packing of the title structure (b) and the monohydrate (c), viewed paralell to the stacking vector of the common SC; one instance of the SC is highlighted in each strutcure. Hydrogen bonds are indicated by broken lines, H atoms are omitted for clarity, Cl and water O atoms are drawn as balls.

## supplementary crystallographic information

### Comment

Morphine is the principal alkaloid of opium. Several crystal forms of the free base as well as salts have been investigated in previous studies: a monohydrate (Bye, 1976), a hydrochloride trihydrate (Gylbert, 1973), a hydroiodide dihydrate (Mackay & Hodgkin, 1955), a complex with β–phenylhydracrylic acid (Lutz & Spek, 1998) and a bis(morphininium) dihydrogensulfate pentahydrate (Wongweichintana *et al.*, 1984). The crystal structure of the title salt was previously solved from powder data by Guguta *et al.* (2008). However, the corresponding atomic coordinates are not available from the Cambridge Structural Database (Allen, 2002) or from supplementary materials accompanying this report.

The asymmetric unit of the title salt contains of one formula unit (Figure 1). The geometry of the molecular morphine scaffold with its five rings agrees with the characteristics of the previously investigated salt and free base structures. The morphinium cation of the title salt is doubly O—H···Cl bonded to the anion so that a *R*^1^_2_(10) ring (Etter *et al.*, 1990; Bernstein *et al.*, 1995) is formed. The Cl^-^ ion is additionally N—H···Cl bonded to the protonation site of a second cation, which is related to the first cation by a 2_1_ screw operation parallel to the *b*–axis. An infinite hydrogen–bonded chain is formed as a result of these interactions, and the chain structure propagates parallel to the *b*-axis (Figure 2*a*). The closest intermolecular C—H···O and C—H···π contacts are listed in Table 2 and shown in Figure 2 *b*.

The program *XPac* (Gelbrich & Hursthouse, 2005) was used to compare the packing of the morphinium ions with that of the analogous moieties in the five related structures mentioned above (Bye, 1976; Gylbert, 1973; Mackay & Hodgkin, 1955; Lutz & Spek, 1998; Wongweichintana *et al.*, 1984). In this group, the closest similarity relationship involving the title structure is based on a single stack of molecules ('one–dimensional supramolecular construct', Figure 3) that is also present in the monohydrate (Bye, 1976). In both crystals the corresponding stacking vector lies parallel [100] with *d* = 7.359 Å for the title structure and *d* = 7.438 Å for the monohydrate. The corresponding *XPac* dissimilarity index *x* (Gelbrich *et al.*, 2012) is 8.2, calculated for a cluster comprising a central molecule and its two next neighbours in the stack on the basis of the positions of all 21 non–H atoms. This value confirms that the fundamental geometry of the stack is maintained. However, the geometry of the stack is somewhat affected by its crystal environment, which accomodates the specific hydrogen bond preferences of the second chemical component, *i.e.* Cl^-^ in the case of the title structure and H_2_O in the monohydrate.

### Experimental

The investigated compound was obtained from Heilmittelwerke Wien, Austria. Block-shaped crystals of the title compound were produced by slow evaporation from an ethanol solution.

### Refinement

The H atoms were identified in a difference map. Methyl H atoms were idealized and included as rigid groups allowed to rotate but not tip (C—H = 0.98 Å). H atoms bonded to tertiary CH (C—H = 0.99 Å), secondary CH_2_ (C—H = 0.99 Å) and aromatic carbon atoms (C—H = 0.95 Å) were positioned geometrically. Hydrogen atoms attached to O and N atoms were refined with restrained distances [O—H = 0.82 (1) Å] and [N—H = 0.93 (1) Å]. The parameters *U*_iso_ of all H atoms were refined freely.

### Crystal data

**Table:**

C_17_H_20_NO_3_^+^·Cl^−^	*F*(000) = 680
*M**_r_* = 321.79	*D*_x_ = 1.411 Mg m^−^^3^
Orthorhombic, *P*2_1_2_1_2_1_	Mo *K*α radiation, λ = 0.7107 Å
Hall symbol: P 2ac 2ab	Cell parameters from 4272 reflections
*a* = 7.3504 (2) Å	θ = 3.0–29.2°
*b* = 12.8524 (5) Å	µ = 0.27 mm^−^^1^
*c* = 16.0372 (5) Å	*T* = 173 K
*V* = 1515.04 (9) Å^3^	Block, colourless
*Z* = 4	0.20 × 0.20 × 0.20 mm

### Data collection

**Table:**

Oxford Diffraction Xcalibur (Ruby, Gemini ultra) diffractometer	2971 independent reflections
Radiation source: Enhance (Mo) X-ray Source	2803 reflections with *I* > 2σ(*I*)
Graphite monochromator	*R*_int_ = 0.024
Detector resolution: 10.3575 pixels mm^-1^	θ_max_ = 26.0°, θ_min_ = 3.0°
ω scans	*h* = −9→9
Absorption correction: multi-scan (*CrysAlis PRO*; Oxford Diffraction, 2003)	*k* = −12→15
*T*_min_ = 0.982, *T*_max_ = 1.000	*l* = −19→19
7406 measured reflections	

### Refinement

**Table:**

Refinement on *F*^2^	Secondary atom site location: difference Fourier map
Least-squares matrix: full	Hydrogen site location: difference Fourier map
*R*[*F*^2^ > 2σ(*F*^2^)] = 0.027	H atoms treated by a mixture of independent and constrained refinement
*wR*(*F*^2^) = 0.069	*w* = 1/[σ^2^(*F*_o_^2^) + (0.0324*P*)^2^ + 0.2844*P*] where *P* = (*F*_o_^2^ + 2*F*_c_^2^)/3
*S* = 1.04	(Δ/σ)_max_ = 0.001
2971 reflections	Δρ_max_ = 0.18 e Å^−^^3^
229 parameters	Δρ_min_ = −0.15 e Å^−^^3^
3 restraints	Absolute structure: Flack (1983), 1245 Friedel pairs
Primary atom site location: structure-invariant direct methods	Flack parameter: 0.02 (5)

### Special details

**Table:**

Geometry. All e.s.d.'s (except the e.s.d. in the dihedral angle between two l.s. planes) are estimated using the full covariance matrix. The cell e.s.d.'s are taken into account individually in the estimation of e.s.d.'s in distances, angles and torsion angles; correlations between e.s.d.'s in cell parameters are only used when they are defined by crystal symmetry. An approximate (isotropic) treatment of cell e.s.d.'s is used for estimating e.s.d.'s involving l.s. planes.
Refinement. Refinement of *F*^2^ against ALL reflections. The weighted *R*-factor *wR* and goodness of fit *S* are based on *F*^2^, conventional *R*-factors *R* are based on *F*, with *F* set to zero for negative *F*^2^. The threshold expression of *F*^2^ > σ(*F*^2^) is used only for calculating *R*-factors(gt) *etc*. and is not relevant to the choice of reflections for refinement. *R*-factors based on *F*^2^ are statistically about twice as large as those based on *F*, and *R*- factors based on ALL data will be even larger.

### Fractional atomic coordinates and isotropic or equivalent isotropic displacement parameters (Å^2^)

**Table:**

	*x*	*y*	*z*	*U*_iso_*/*U*_eq_	
Cl1	0.96273 (5)	0.72090 (4)	0.82895 (3)	0.03438 (12)	
O1	0.83825 (19)	0.64336 (10)	0.65098 (8)	0.0367 (3)	
H1O	0.877 (3)	0.6586 (18)	0.6972 (8)	0.051 (7)*	
O2	0.55524 (15)	0.77200 (8)	0.73074 (6)	0.0242 (2)	
O3	0.62377 (17)	0.85259 (10)	0.88464 (7)	0.0294 (3)	
H3O	0.709 (2)	0.8207 (16)	0.8625 (13)	0.050 (7)*	
N1	0.29227 (19)	1.08986 (11)	0.56252 (9)	0.0242 (3)	
H1N	0.221 (2)	1.1239 (15)	0.6013 (10)	0.044 (6)*	
C1	0.7896 (2)	0.86915 (13)	0.51135 (9)	0.0243 (4)	
H1	0.8436	0.8943	0.4615	0.042 (6)*	
C2	0.8430 (2)	0.77357 (14)	0.54336 (10)	0.0259 (4)	
H2	0.9308	0.7338	0.5139	0.031 (5)*	
C3	0.7717 (2)	0.73423 (12)	0.61752 (10)	0.0245 (3)	
C4	0.6342 (2)	0.79226 (12)	0.65458 (9)	0.0202 (3)	
C5	0.5554 (2)	0.92311 (12)	0.82414 (9)	0.0227 (3)	
H5	0.4628	0.9669	0.8534	0.019 (4)*	
C6	0.4532 (2)	0.86543 (12)	0.75454 (9)	0.0202 (3)	
H6	0.3301	0.8446	0.7753	0.022 (4)*	
C7	0.6973 (2)	0.99708 (13)	0.79168 (10)	0.0242 (4)	
H7	0.8131	1.0002	0.8179	0.031 (5)*	
C8	0.6628 (2)	1.05776 (13)	0.72718 (10)	0.0232 (4)	
H8	0.7501	1.1073	0.7090	0.027 (5)*	
C9	0.4862 (2)	1.09416 (13)	0.59477 (10)	0.0220 (3)	
H9	0.5211	1.1691	0.5999	0.022 (4)*	
C10	0.6209 (2)	1.04229 (14)	0.53414 (11)	0.0272 (4)	
H10A	0.5727	1.0489	0.4767	0.029 (5)*	
H10B	0.7377	1.0805	0.5364	0.048 (6)*	
C11	0.6574 (2)	0.92863 (13)	0.55164 (9)	0.0207 (3)	
C12	0.5743 (2)	0.88337 (12)	0.61978 (9)	0.0188 (3)	
C13	0.43158 (19)	0.93188 (12)	0.67453 (9)	0.0181 (3)	
C14	0.4832 (2)	1.04739 (12)	0.68285 (9)	0.0193 (3)	
H14	0.3875	1.0836	0.7163	0.012 (4)*	
C15	0.2383 (2)	0.92357 (13)	0.63760 (10)	0.0243 (4)	
H15A	0.2078	0.8493	0.6290	0.033 (5)*	
H15B	0.1498	0.9528	0.6778	0.017 (4)*	
C16	0.2217 (2)	0.98094 (13)	0.55534 (11)	0.0283 (4)	
H16A	0.2912	0.9433	0.5119	0.030 (5)*	
H16B	0.0924	0.9827	0.5380	0.021 (4)*	
C17	0.2666 (3)	1.14956 (15)	0.48358 (11)	0.0349 (4)	
H17A	0.3241	1.1120	0.4374	0.035 (5)*	
H17B	0.3225	1.2184	0.4892	0.046 (6)*	
H17C	0.1363	1.1574	0.4723	0.041 (6)*	

### Atomic displacement parameters (Å^2^)

**Table:**

	*U*^11^	*U*^22^	*U*^33^	*U*^12^	*U*^13^	*U*^23^
Cl1	0.0245 (2)	0.0376 (2)	0.0411 (2)	0.00496 (19)	0.00355 (18)	0.0024 (2)
O1	0.0476 (8)	0.0244 (7)	0.0383 (8)	0.0127 (6)	0.0048 (6)	−0.0009 (6)
O2	0.0310 (6)	0.0171 (5)	0.0246 (6)	0.0019 (5)	0.0063 (5)	0.0017 (4)
O3	0.0345 (7)	0.0337 (7)	0.0201 (6)	0.0081 (6)	0.0012 (5)	0.0051 (5)
N1	0.0265 (7)	0.0226 (7)	0.0236 (7)	0.0017 (6)	−0.0054 (6)	0.0008 (6)
C1	0.0234 (8)	0.0297 (9)	0.0199 (8)	−0.0052 (7)	0.0032 (7)	−0.0035 (7)
C2	0.0240 (8)	0.0281 (9)	0.0256 (8)	0.0007 (8)	0.0028 (7)	−0.0120 (8)
C3	0.0274 (8)	0.0180 (8)	0.0282 (8)	0.0014 (7)	−0.0008 (7)	−0.0051 (7)
C4	0.0222 (7)	0.0190 (8)	0.0195 (7)	−0.0047 (7)	0.0005 (6)	−0.0038 (6)
C5	0.0258 (8)	0.0242 (8)	0.0181 (7)	0.0037 (7)	0.0012 (7)	0.0008 (6)
C6	0.0201 (7)	0.0184 (8)	0.0221 (7)	0.0003 (7)	0.0049 (7)	0.0008 (6)
C7	0.0236 (8)	0.0250 (8)	0.0240 (8)	−0.0018 (7)	−0.0044 (7)	−0.0058 (7)
C8	0.0245 (8)	0.0185 (8)	0.0267 (8)	−0.0063 (7)	−0.0010 (7)	−0.0032 (7)
C9	0.0235 (8)	0.0168 (7)	0.0258 (8)	−0.0025 (7)	−0.0040 (6)	0.0029 (6)
C10	0.0279 (9)	0.0268 (9)	0.0269 (9)	0.0001 (8)	0.0029 (7)	0.0075 (7)
C11	0.0201 (8)	0.0243 (8)	0.0177 (7)	−0.0042 (7)	−0.0020 (6)	−0.0009 (6)
C12	0.0188 (7)	0.0179 (7)	0.0198 (7)	−0.0030 (6)	−0.0024 (6)	−0.0041 (6)
C13	0.0173 (7)	0.0177 (7)	0.0192 (7)	−0.0029 (6)	−0.0002 (6)	0.0002 (6)
C14	0.0204 (7)	0.0167 (7)	0.0209 (8)	−0.0008 (6)	−0.0001 (6)	−0.0016 (6)
C15	0.0200 (8)	0.0225 (8)	0.0303 (9)	−0.0033 (7)	−0.0022 (7)	−0.0012 (7)
C16	0.0256 (9)	0.0268 (9)	0.0325 (9)	−0.0009 (7)	−0.0096 (7)	−0.0051 (8)
C17	0.0449 (11)	0.0311 (10)	0.0288 (9)	0.0037 (9)	−0.0109 (8)	0.0055 (8)

### Geometric parameters (Å, º)

**Table:**

O1—C3	1.375 (2)	C7—H7	0.9500
O1—H1O	0.817 (9)	C8—C14	1.505 (2)
O2—C4	1.3770 (17)	C8—H8	0.9500
O2—C6	1.4665 (18)	C9—C14	1.535 (2)
O3—C5	1.4196 (19)	C9—C10	1.540 (2)
O3—H3O	0.831 (10)	C9—H9	1.0000
N1—C17	1.492 (2)	C10—C11	1.511 (2)
N1—C16	1.497 (2)	C10—H10A	0.9900
N1—C9	1.517 (2)	C10—H10B	0.9900
N1—H1N	0.925 (9)	C11—C12	1.380 (2)
C1—C2	1.388 (2)	C12—C13	1.503 (2)
C1—C11	1.395 (2)	C13—C14	1.538 (2)
C1—H1	0.9500	C13—C15	1.543 (2)
C2—C3	1.395 (2)	C14—H14	1.0000
C2—H2	0.9500	C15—C16	1.516 (2)
C3—C4	1.390 (2)	C15—H15A	0.9900
C4—C12	1.370 (2)	C15—H15B	0.9900
C5—C7	1.504 (2)	C16—H16A	0.9900
C5—C6	1.536 (2)	C16—H16B	0.9900
C5—H5	1.0000	C17—H17A	0.9800
C6—C13	1.549 (2)	C17—H17B	0.9800
C6—H6	1.0000	C17—H17C	0.9800
C7—C8	1.320 (2)		
			
C3—O1—H1O	105.8 (17)	C10—C9—H9	107.7
C4—O2—C6	106.96 (11)	C11—C10—C9	114.53 (14)
C5—O3—H3O	106.9 (16)	C11—C10—H10A	108.6
C17—N1—C16	111.81 (13)	C9—C10—H10A	108.6
C17—N1—C9	112.91 (14)	C11—C10—H10B	108.6
C16—N1—C9	112.69 (13)	C9—C10—H10B	108.6
C17—N1—H1N	104.8 (14)	H10A—C10—H10B	107.6
C16—N1—H1N	107.3 (14)	C12—C11—C1	116.35 (15)
C9—N1—H1N	106.7 (13)	C12—C11—C10	118.42 (14)
C2—C1—C11	120.71 (15)	C1—C11—C10	124.55 (15)
C2—C1—H1	119.6	C4—C12—C11	122.73 (14)
C11—C1—H1	119.6	C4—C12—C13	109.92 (13)
C1—C2—C3	122.00 (15)	C11—C12—C13	126.61 (14)
C1—C2—H2	119.0	C12—C13—C14	106.19 (12)
C3—C2—H2	119.0	C12—C13—C15	112.92 (13)
O1—C3—C4	123.21 (14)	C14—C13—C15	109.10 (12)
O1—C3—C2	120.45 (15)	C12—C13—C6	100.58 (12)
C4—C3—C2	116.33 (15)	C14—C13—C6	115.78 (12)
C12—C4—O2	112.80 (13)	C15—C13—C6	111.97 (12)
C12—C4—C3	121.21 (14)	C8—C14—C9	112.77 (13)
O2—C4—C3	125.76 (14)	C8—C14—C13	110.04 (13)
O3—C5—C7	113.23 (13)	C9—C14—C13	107.56 (12)
O3—C5—C6	111.20 (13)	C8—C14—H14	108.8
C7—C5—C6	113.13 (12)	C9—C14—H14	108.8
O3—C5—H5	106.2	C13—C14—H14	108.8
C7—C5—H5	106.2	C16—C15—C13	112.00 (13)
C6—C5—H5	106.2	C16—C15—H15A	109.2
O2—C6—C5	109.51 (12)	C13—C15—H15A	109.2
O2—C6—C13	106.75 (11)	C16—C15—H15B	109.2
C5—C6—C13	112.69 (12)	C13—C15—H15B	109.2
O2—C6—H6	109.3	H15A—C15—H15B	107.9
C5—C6—H6	109.3	N1—C16—C15	111.09 (13)
C13—C6—H6	109.3	N1—C16—H16A	109.4
C8—C7—C5	120.73 (15)	C15—C16—H16A	109.4
C8—C7—H7	119.6	N1—C16—H16B	109.4
C5—C7—H7	119.6	C15—C16—H16B	109.4
C7—C8—C14	119.11 (15)	H16A—C16—H16B	108.0
C7—C8—H8	120.4	N1—C17—H17A	109.5
C14—C8—H8	120.4	N1—C17—H17B	109.5
N1—C9—C14	106.60 (12)	H17A—C17—H17B	109.5
N1—C9—C10	111.91 (13)	N1—C17—H17C	109.5
C14—C9—C10	114.89 (13)	H17A—C17—H17C	109.5
N1—C9—H9	107.7	H17B—C17—H17C	109.5
C14—C9—H9	107.7		

### Hydrogen-bond geometry (Å, º)

Cg1 is the centroid of the C1–C4/C12/C11 benzene ring.

**Table:**

*D*—H···*A*	*D*—H	H···*A*	*D*···*A*	*D*—H···*A*
O1—H1*O*···Cl1	0.82 (1)	2.35 (1)	3.1585 (14)	173 (2)
O3—H3*O*···Cl1	0.83 (1)	2.32 (1)	3.1416 (13)	168 (2)
N1—H1*N*···Cl1^i^	0.93 (1)	2.15 (1)	3.0626 (15)	169 (2)
C17—H17*C*···O3^ii^	0.98	2.38	3.278 (2)	153
C14—H14···O2^i^	1.00	2.60	3.2149 (19)	120
C2—H2···*Cg*1^iii^	0.95	2.71	3.638 (2)	165

Symmetry codes: (i) −*x*+1, *y*+1/2, −*z*+3/2; (ii) −*x*+1/2, −*y*+2, *z*−1/2; (iii) *x*+1/2, −*y*+3/2, −*z*+1.
